# Vibrotactile Feedback Strategies for Trunk-Stabilizing Exercises in a Home-Based Scenario: Qualitative Interview Study Among Physiotherapists

**DOI:** 10.2196/62903

**Published:** 2025-07-08

**Authors:** Kaya Holzmeyer, Lisa-Marie Lüneburg, Luca Oppici, Philipp Flößel, Doris Lachmann, Jens Krzywinski, Susanne Narciss

**Affiliations:** 1 Psychology of Learning and Instruction, Faculty of Psychology, Technische Universität Dresden, Dresden, Germany; 2 Industrial Design Engineering, Faculty of Mechanical Engineering, Technische Universität Dresden, Helmholtzstr. 10, Dresden, 01062, Germany, 49 351-463-35761; 3 Cluster of Excellence CeTI: "Centre for Tactile Internet with Human-in-the-Loop", Technische Universität Dresden, Dresden, Germany; 4 The Department of Teacher Education and Outdoor Studies, Norwegian School of Sport Sciences, Oslo, Norway; 5 Center of Orthopedic, Trauma and Plastic Surgery, University Hospital Carl Gustav Carus, Medical Faculty Carl Gustav Carus, Technische Universität Dresden, Dresden, Germany

**Keywords:** vibrotactile feedback, physiotherapy, home exercises, motor learning, Internet of Things, haptic, wearable, qualitative research, user-centered design, digital health, back pain

## Abstract

**Background:**

Physiotherapy treatments frequently incorporate a combination of hands-on, therapist-led exercises and hands-off, home-based exercises, whereby patients perform exercises independently. A key challenge in home-based rehabilitation is providing patients with effective feedback to guide their movements. A range of strategies is being reviewed to address this issue and a starting point for developing effective and implementable strategies to gather feedback requirements from the end users is also being considered.

**Objective:**

This study aims to derive requirements for designing a vibrotactile feedback strategy for a wearable device for physiotherapy home exercises. To achieve this, it is essential to consider the principles of feedback design as set forth by the field of instructional psychology and to involve physiotherapists in the process.

**Methods:**

The feedback behavior of 9 physical therapists was observed during a staged training scenario. Guided interviews were conducted to explore their recommendations for a vibrotactile feedback system. Observational data and interviews were analyzed using a combined deductive and inductive category system. For each exercise-specific motion pattern, it was recorded whether feedback was provided. Instances of feedback were systematically coded according to feedback modality, timing, and content; haptic feedback was additionally categorized by localization. Interview statements referring to the use of vibrotactile feedback were categorized by modality, task requirements, localization, time, content, frequency, function, and individualization. Quantitative data from observations were evaluated using frequency distributions, and qualitative interview data were analyzed using content structuring content analysis.

**Results:**

On average, 50.2% (SD 13.4%) of the observed feedback content provided by each therapist consisted of the presentation of correct exercise execution. A smaller proportion was exclusively confirmatory feedback or the additional provision of elaborated information. Therapists provided feedback in 57.3% (SD 16.3%) of the movement repetitions, on average, while no feedback was given in 42.7% (SD 16.3%) of the repetitions. The interview data supported a reduction in feedback. Regarding feedback timing, 70.8% (SD 13.1%) of the feedback observations were given concurrently with the task across therapists; the proportion of feedback given after the exercise execution was smaller. On average, across therapists, 51.9% (SD 12.1%) of the feedback was auditory, and a further proportion was multimodal with an auditory component. Regarding feedback localization, haptic feedback was often given proximal to the trunk, in the knee and foot region. Frequent combinations of the categories were auditory, simple confirmatory feedback, often given when terminal, or the presentation of the correct exercise execution given auditorily or multimodally concurrent to exercise execution.

**Conclusions:**

This study offers insights into the design of a vibrotactile feedback strategy. We identified key feedback characteristics recommended by physical therapists: reduced frequency, differentiated content, multimodal feedback, and trunk-proximal vibrotactile cues. These approaches should be tested in hypotheses-driven research and further patient studies should be conducted.

## Introduction

### Background

Feedback is a key component in physiotherapy, playing a crucial role in achieving therapy goals [1,2]. Physiotherapists provide hands-on feedback during treatment, a form that is lacking for patients following an at-home exercise regime. The use of home exercises has seen a notable rise in recent years. This growth can be attributed to 2 key factors: the effectiveness of active methods over passive treatment approaches and the shortage of health care professionals [3]. Standard technologies include apps and devices that provide visual or auditory feedback [4-11], with some using gamification approaches [12,13]. Recent advances in tactile internet technologies (tactile internet with human in the loop) have opened up new possibilities for manipulating and controlling virtual or real objects with almost no latency, ensuring minimal delay in data transmission. These technologies allow for real-time tactile feedback, which has been shown to positively impact motor skill performance and learning [14-19]. Such feedback is increasingly used in rehabilitation, offering innovative approaches to physical therapy [20-23]. Specifically, wearables, which are devices that embed technology into garments or accessories directly worn close to the skin, such as shirts, bracelets, watches, anklets, or waistcoats, are suitable for those purposes. These devices use vibration actuators to deliver tactile feedback to the wearer while using sensors to monitor and classify movement data during exercises. In the context of home-based physiotherapy, external tactile feedback offers a promising approach to address the absence of hands-on guidance. This tactile feedback can be delivered through wearable systems. This study is part of a broader effort to develop a haptic physiotherapy assistant, including a wearable device that provides vibrotactile feedback. Among the wearable formats, the shirt stands out as particularly promising due to its ability to cover larger areas of the body, allowing for precise and distributed feedback during rehabilitation exercises.

Recent reviews highlight both the potential of these wearable technologies for home-based rehabilitation and the need for empirically driven strategies to optimize feedback mechanisms [24,25]. By addressing these gaps, this study aims to establish a robust foundation for designing and implementing effective haptic feedback systems for physiotherapy practice.

Therefore, challenges from the perspective of instructional psychology and interaction design are considered when developing a vibrotactile feedback strategy. The interactive tutoring feedback (ITF) model, which originated in instructional psychology, posits that feedback information can be sourced from 2 distinct domains: the internal domain, which encompasses information generated internally by the feedback recipient, such as proprioception, and the external domain, which encompasses feedback originating from external sources, such as wearables [26,27]. A haptic physiotherapy assistant, which is targeted by this research project, is an external feedback source. It is crucial to design the external feedback in a manner that does not override the internal feedback of the feedback recipients. As internal feedback is available to the feedback recipient in the long term, external feedback should be designed in such a way that the recipient is supported in generating internal feedback. This approach minimizes dependence on the external system and facilitates long-term learning [26-28]. Therefore, this study follows a strategic approach to design effective external feedback, that is, a coordinated plan that implies decisions about specific feedback properties [26,27]. A feedback strategy integrates decisions about at least the following properties:

Which content should be provided by the feedback? Feedback always provides a certain type of evaluative information (eg, knowledge of the result in terms of a single confirmation or rejection or knowledge of the correct result). Furthermore, it can provide additional, elaborated information components. Elaborated components can offer information about task requirements, conceptual knowledge, error type, learning process, or strategies, that is, information supporting the feedback recipient in further knowledge-gaining or learning process, or a combination of these components [26].

How is the feedback content presented using the formal and technical design properties that are available in a task context? Designing the presentation of the feedback contents includes making decisions on feedback timing, scheduling, frequency, and the feedback modality. Concerning the feedback timing, literature differentiates between concurrent feedback, that is, information delivered during task execution, and terminal feedback, that is, information that is either delivered immediately or with delay after finishing a task. Feedback can be presented continuously after each event or intermittently with a reduced frequency, such as fading-in or fading-out strategies. A technical feedback system can provide feedback in a unimodal vibrotactile way or in a multimodal way by supplementing, for example, vibrotactile feedback with other modalities.

A challenge in the design process is the early integration of potential users. Human-centered design aims to increase the usability of end products. An important goal is to promote and improve the understanding of tasks, contexts, and users, as well as the resulting requirements [29-31]. This work is settled in the early phase of the development of a wearable feedback system, the unstructured and uncertain fuzzy front-end. So, human-centered design could be an effective method of improving the design process [31].

To the best of our knowledge, no extant literature addresses the characteristics of tactile feedback in the context of physiotherapy home exercises, highlighting the need for further investigation.

### Objectives

The objective of this study is to derive requirements for effective external vibrotactile feedback for home exercises in physiotherapy. Specifically, the investigation will encompass the characteristics that, following the principles of instructional psychology, must be considered during the design process to ensure the effectiveness of external feedback (modality, content, and timing) [26-28]. Concurrently, we are integrating feedback behavior (modality, content, timing, and localization) from physiotherapists to understand the context of use.

## Methods

### Overview

We conducted a qualitative study to gain a fundamental understanding of physiotherapists’ feedback behavior when providing home-based exercise instructions. The reporting and writing of this study were guided by the standards for reporting qualitative research and the COREQ (Consolidated Criteria for Reporting Qualitative Research) checklist [32,33] (Checklist 1). The data collection was structured in 2 parts. In the first part, we observed how physical therapists delivered feedback to a standardized patient in a typical hands-on scenario. In the second part, we asked the physical therapist to recommend the most appropriate use of a wearable device to provide feedback to a standardized patient in a hands-off home-based scenario. The data was collected using video-based participant observation and guided interviews. We staged a training scenario with home exercises for low back pain. The study was conducted over the period from February to March 2022.

### Participants

Physical therapists working in physiotherapy facilities in Dresden, Germany, were recruited as study participants. A purposive sample of 30 public physiotherapy facilities was initially contacted via email and telephone to assess their interest in study participation. All facilities offered training therapy or active physiotherapy, as indicated on their websites. At the initial point of contact, which was conducted for a specific purpose and by the protocol for engaging with public institutions, physiotherapists were informed about this study’s aim, procedures, and data collection. Physiotherapists were invited to participate after confirming their interest. This recruitment procedure was authorized by the Data Protection Officer of TU Dresden, as required by § 12 (1) of the Saxon Data Protection Implementation Act, with no alternative data collection methods identified. The planned inclusion criteria of participants were at least 3 years of professional experience as a physiotherapist and expertise in the treatment of home exercises. The investigator and participants were not previously acquainted, except for the initial recruitment contact.

### Ethical Considerations

This study was approved by the Ethics Committee of Technische Universität Dresden (SR-EK-529112021) and was conducted in accordance with the principles of the Declaration of Helsinki.

The research involved human participants and therefore underwent full ethical review. This study’s design, including procedures for data collection via video recording, was assessed and approved under the stated institutional review board protocol. No exemptions or waivers were sought. The approval documentation is available upon request.

Before participation, all individuals were provided with detailed written information about this study’s aims, procedures, data handling practices, and their rights, including the right to withdraw at any time without penalty. Written informed consent was obtained from all participants. The consent process explicitly addressed the use of video data and the scope of data processing involved.

All data collected during this study were either anonymized or deidentified to protect participant privacy. In cases where full anonymization was not feasible due to the nature of the video recordings, strict access controls and data encryption were implemented to safeguard the information. Data access was restricted to authorized research personnel only.

Participants were compensated €15 (US $17.15) for their time and any inconvenience incurred due to their involvement in this study, including potential work time lost. Compensation was not contingent on completion of this study.

### Staged Training and Interview

**Figure 1.**
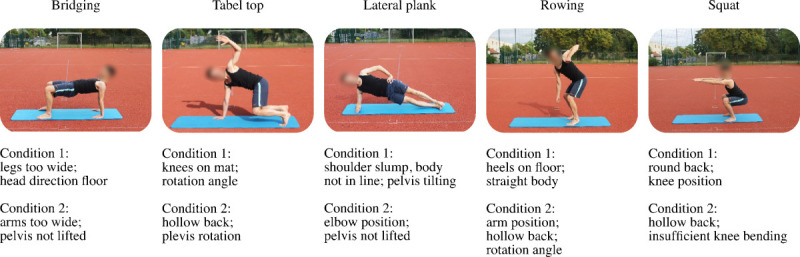
Overview of home exercises and predefined error conditions used during the staged training session.

The staged training session consisted of 5 home exercises for the treatment of nonspecific low back pain. We preselected home exercises based on a multicenter controlled trial [34]. Following a systematic selection process, 3 physiotherapists evaluated and selected effective and commonly used exercises through interviews. The physiotherapists added additional exercises for daily use to ensure practical applicability. We extracted requirements and 20 typical errors for the 5 exercises. To ensure comparability of the exercises and the physiotherapists’ feedback behavior, we trained 1 healthy individual to perform the exercises in both correct and error conditions. This approach allowed for a reproducible staged training session with a standardized patient (Figure 1). A detailed description of the staged training is provided in Multimedia Appendix 1. We obtained an explicit consent from the individual in Figure 1 as well as in Multimedia Appendix 1 to use their image for publication.

Interview guide development was grounded in the ITF model, which describes the influencing factors, interdependencies and effects of feedback in competence acquisition and can thus form the basis for the development of effective feedback strategies [26,27]. The semistructured interview mainly contained open-ended questions and was supplemented by individual closed-ended questions (Table 1). The order and phrasing of the questions were flexible and could be adapted to the course of the interview. To be able to go into more detail about unforeseen, thematically relevant statements, additional ad hoc questions were asked. The interview lasted approximately 15 minutes and was audio-recorded. Notes and photos were taken to supplement the audio information, for example, when a participant visualized a statement.

**Table 1.** Semistructured interview schedule.

Topic	Questions and prompts
Task requirements	Please describe a possible behavior that could initiate vibrotactile feedback.
Localization	Besides the body localization you already showed, are there other body parts where vibrotactile feedback could be delivered?
Timing	To schedule the feedback to movement repetitions: when should vibrotactile feedback be presented?
Frequency	How often should vibrotactile feedback be delivered? Within a single training session? Within the training period? Which technique of reducing frequency is effective?
Content	Which information should be given by vibrotactile feedback?
Individualization	Which feedback properties should be adaptable to individuals?

A preliminary pilot study was conducted in an unpublished format before the main study. The piloting was performed with a trained physiotherapist with 10 years of professional experience; he fulfilled the inclusion criteria of the selected sample. The piloting aimed to check the comprehensibility and accuracy of the instructions, questions, interview, and home exercises and to develop them further. Following the piloting phase, we refined the semistructured interview questions and modified the execution of 2 exercises (rowing and squatting). The piloting also allowed us to validate the research design and test its implementation. This process also enhanced the study’s feasibility and confidence for both the standardized patient and the researcher.

### Study Procedure

**Figure 2.**
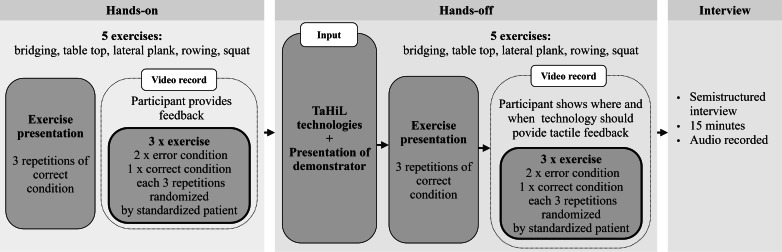
Study procedure with 3 main phases of data collection. TaHiL: tactile internet with human in the loop.

This study took place at the participants’ facilities and involved individual observation and interviews (Figure 2).

After completing demographic inquiries, we started the staged training which consists of a hands-on and hands-off part followed by an interview as mentioned above. Participants were instructed to provide feedback to the standardized patient during the staged training session, deciding independently on the type and timing of feedback. Each exercise began with a correct demonstration to illustrate the target execution, during which no feedback was given. Participants then completed 3 runs of the same exercise: 1 without errors and 2 with errors. Each run consisted of 3 repetitions of a movement pattern or 5 seconds of stabilization. After completing 1 exercise, participants moved on to the next, performing it across 3 feedback runs. When participants provided feedback, the standardized patient responded by performing additional repetitions or maintaining stabilization in the corrected form, explicitly addressing the feedback without correcting unmentioned errors.

**Figure 3.**
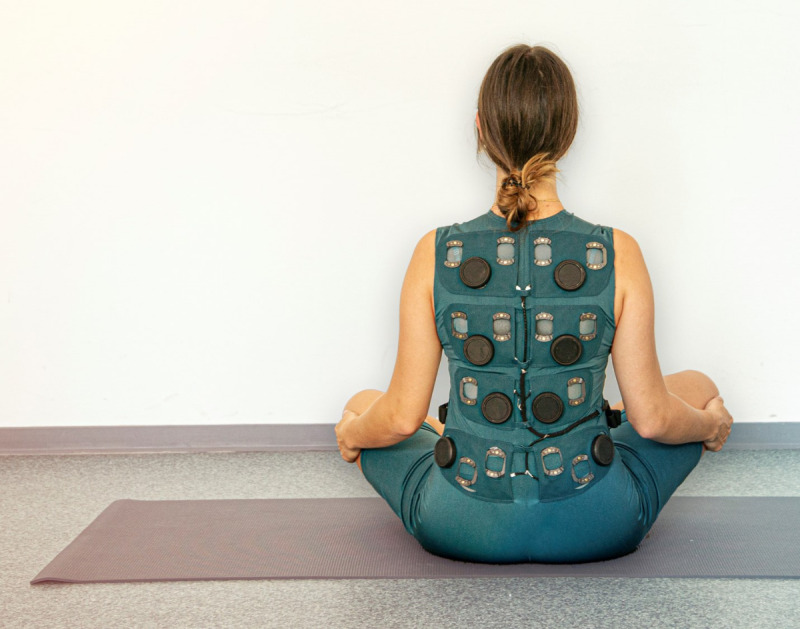
Demonstrator of wearable haptic physiotherapy assistant.

After finishing the hands-on part, participants were informed about tactile internet with human in the loop interventions and a simple demonstrator of a possible wearable feedback device was presented (Figure 3). This assistant is a wearable device—a body-worn cyber-physical system designed to enable advanced multimodal human-machine interactions through vibrotactile feedback. The envisioned system provides real-time haptic feedback through vibrations on specific body parts where a posture needs to be corrected during the exercise. Furthermore, the concept entails an AI-supported expert system with embedded sensors to classify movement data during exercises, triggering the vibrotactile feedback when deviations from the intended movement pattern are detected. This technology can provide intuitive, hands-free guidance and support by incorporating vibrotactile stimulation into clothing.

The authors instructed them to imagine a wearable device, similar to the one shown in Figure 3, capable of generating vibrations anywhere on the body (also on arms and legs) in response to specific situations that they felt required feedback. They should imagine a hands-free scenario in which the standardized patient uses such a device independently at home while performing their exercises. Subsequently, they were asked to indicate where and when a tactile feedback system should provide feedback while the standardized patient repeated the entire staged training session. Two video cameras recorded the staged training sessions for further analysis.

### Data Analysis

We transcribed the audio recordings (interviews) with MAXQDA Plus 2022 (release 22.2.0; MAXQDA - Distribution by VERBI GmbH) using a content semantic transcription [35]. The aim of this data analysis was a systematic comparison as well as an exploration of feedback properties. To systematically compare and explore feedback properties, we conducted a content-structuring content analysis, the core of which is a coding system [36]. The main and subcategories of the coding system were developed deductively based on the ITF model [26,27] and prior empirical work [2]. For each category, we created names, content descriptions, examples, and decision rules. Using data from 2 participants (videos and audio transcripts), we performed a trial coding, adding subcategories inductively through subsumption [37]. The same coder repeated the trial coding after 4 weeks, and the agreement between these 2 codings was 87.85% (κ=0.88). A κ value of κ=0.88 is interpreted as a strong agreement in the literature [38]. Additionally, the category system was reviewed and discussed with a research team member. After this trial’s coding, definitions of main and subcategories were modified, for example, if segments could not be clearly assigned to a category or if segments were assigned to different categories when coded again. The main analysis was conducted using a complete and modified category system. The entire category system, including the definitions and examples, is provided in Multimedia Appendix 2. The videos (hands-on and hands-off observations) and audio transcripts (interviews) were segmented and coded.

**Figure 4.**
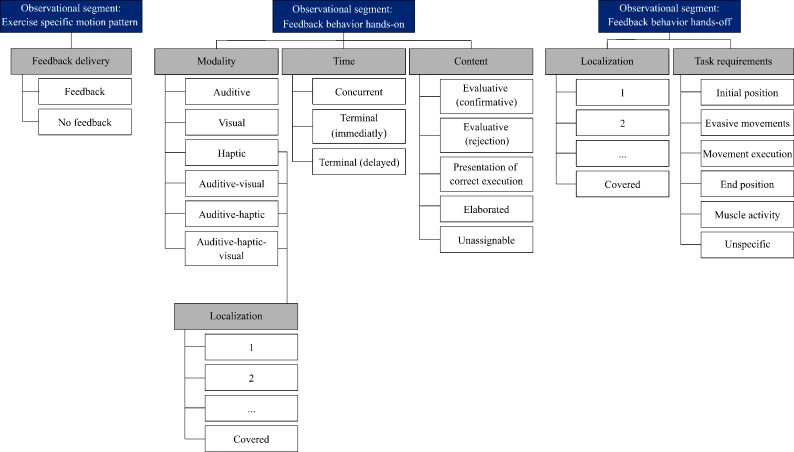
Category system for observed feedback in hands-on context (segmented motion patterns annotated with corresponding feedback events, categorized by modality, timing, content, and localization [body areas 1‐32]) and in hands-off context (feedback instances categorized by localization and task requirements).

In the analysis of the hands-on observations, the exercise-specific motion pattern served as the first observation segment for feedback delivery and was categorized as either “feedback given” or “no feedback given.” Each identified feedback was systematically coded into the following subcategories: feedback modality, timing, and content. Additionally, each haptic feedback was assigned to the feedback localization category (Figure 4). In the analysis of the hands-off observations, the behavioral sequences that were relevant were those associated with the use of a vibrotactile feedback system. Touches that presented information for using vibrotactile feedback systems and statements that referred to using a vibrotactile feedback systems formed relevant coding segments. If a coding segment was detected, it was classified into main and subcategories (Figures4  5).

**Figure 5.**
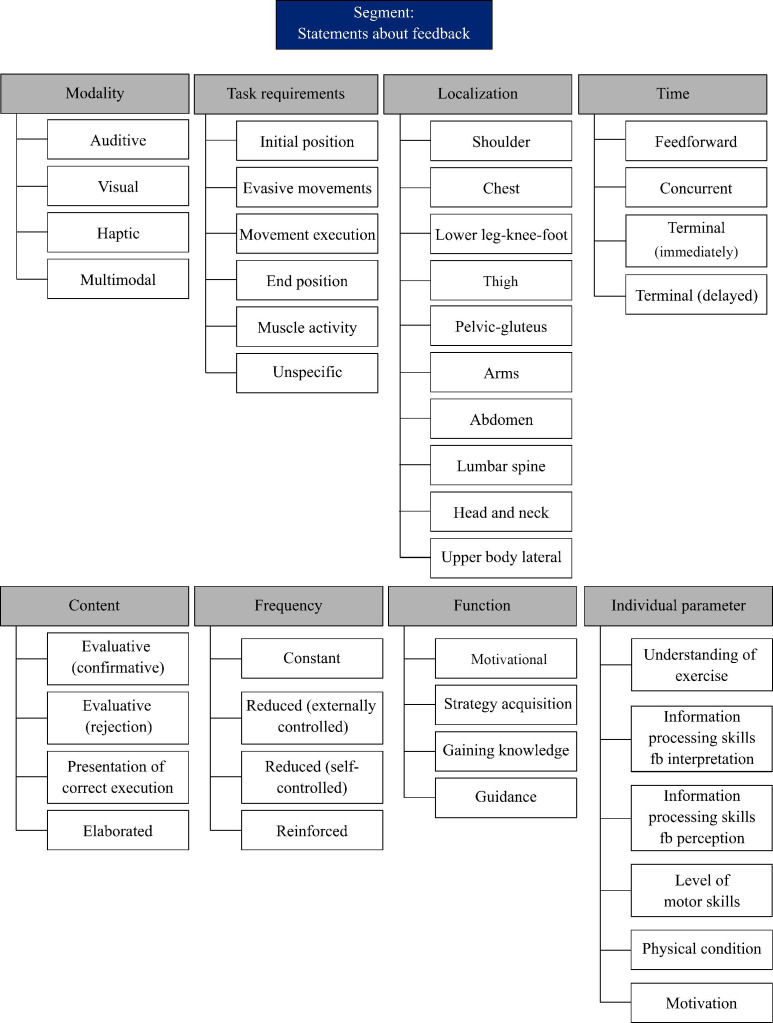
Category system for interview statements on hands-off feedback during staged training: categorized by modality, task requirements, localization, timing, content, frequency, function, and individual parameters. fb: feedback.

We quantified the observational data and calculated the relative frequencies of each subcategory for each participant. For the entire sample, we calculated the means and SDs of each subcategory from the participant-based relative frequencies. To provide a more comprehensive account of feedback behavior, the combination of observed properties of feedback presentation was analyzed. We calculated the relative frequencies of subcategory combinations for each participant. We then calculated the means and SDs of these combinations for the entire sample. We thematically summarized the interview data for each subcategory on a case-by-case basis and analyzed it qualitatively. Additionally, interrelations between the subcategories were analyzed. We analyzed the data using MAXQDA Plus 2022 (release 22.2.0) and R (version 4.2.1; R Foundation).

## Results

### Participant Statistics

Table 2 shows the demographic and occupational characteristics of participants. This study was conducted with a total of 9 participants. Seven were trained physiotherapists, while the others were sport and rehabilitation trainers. The sports therapist and the rehabilitation trainer stated that almost every patient was treated with home exercises. Therefore, we considered them as experienced.

**Table 2.** Participant demographics, occupations, and working experience.

Participant	Education	Working experience (years), n	Treatment with home exercises^a^	Age (years), n	Gender
P1^b^	Physiotherapist	4	Almost every patient	23	Male
P2	Physiotherapist	24	For many patients	52	Male
P3	Physiotherapist	20	Almost every patient	43	Male
P4	Physiotherapist	16	Almost every patient	39	Male
P5	Sport therapist	5	Almost every patient	28	Female
P6	Physiotherapist	1	Almost every patient	26	Female
P7	Rehabilitation trainer	8	Almost every patient	46	Female
P8	Physiotherapist	2.5	Almost every patient	26	Male
P9	Physiotherapist	8	Almost every patient	32	Male

a Possible answers: “almost every patient,” “many patients,” “some patients,” “very few patients.”

b P: participant.

### Feedback Content

As shown in Table 3, on average across therapists 50.2% (SD 13.4%) of observed feedback content was coded as a presentation of correct execution. Elaborated and evaluative feedback was provided in smaller but similar amounts.

Types of evaluative feedback mentioned by 8 of 9 physiotherapists in hands-off context, including nonspecific confirmatory feedback (eg, “Yes, well done”), error signaling (eg, “That’s not fully correct”), and specific confirmatory feedback linked to movement execution. The presentation of correct execution feedback addressed various task requirements, such as muscle activation, spatial movement patterns, initial positioning, movement velocity, and training persistence. The specific task request associated with a particular (haptic) signal would depend on the individual interpretation of the patients.

Four participants made statements about elaborated feedback content. A detailed description of feedback properties named by physiotherapists in hands-off interviews, grouped by theme and linked to task requirements and individual parameters is provided in Multimedia Appendix 3.

### Feedback Frequency

On average, physical therapists provided feedback in 57.3% (SD 16.3%) of repetitions, while no feedback was given in 42.7% (SD 16.3%).

Four participants made statements about elaborated feedback content. A detailed description of feedback properties named by physiotherapists in hands-off interviews, grouped by theme and linked to task requirements and individual parameters is provided in Multimedia Appendix 3.

Interview data revealed unanimous support among participants for reducing feedback frequency (). Reductions were recommended both within individual training sessions and over more extended training periods. Suggested techniques included starting with less feedback and gradually increasing it to refine proprioception, prevent sensory overload, and foster acclimatization in the early stages. Feedback fading aimed to enhance intrinsic body awareness and reduce dependency on external feedback, though no consensus emerged on the optimal fading rate. Suggested initiation periods ranged from 2‐6 weeks.

While most participants supported feedback reduction, 2 highlighted concerns about declining endurance and muscle activity during training. Explicit feedback was recommended for exercise repetitions and signs of muscle fatigue. Feedback frequency was also noted to depend on physical conditions, such as illness or age. In cases of significant error patterns (eg, severe joint overload), immediate corrective feedback was deemed essential. Finally, self-controlled feedback was perceived as less effective for patients lacking intrinsic motivation to improve.

**Table 3.** Observed relative frequencies (%) of feedback properties by category (content, modality, and timing) during staged training in hands-on context (means and SDs).

Feedback propertiemean percentages (SD)s	Mean percentages (SD)
Content	
Evaluative (confirmation)	28.3 (8.4)
Evaluative (rejection)	0 (0)
Presentation correct execution	50.2 (13.4)
Elaborated	21.5 (11.4)
Unassignable	5.6 (1.1)
Modality	
Visual	0 (0)
Auditive	51.9 (12.1)
Haptic	0.8 (1.2)
Auditive or visual	16.4 (16.0)
Auditive or haptic	29.2 (24.4)
Auditive, haptic, or visual	1.9 (2.6)
Timing	
Concurrent	70.8 (13.1)
Terminal immediately	27.1 (13.6)
Terminal delayed	2.3 (4.6)

### Feedback Timing

On average, across therapists, mainly concurrent feedback could be observed (70.8%, SD 13.1%; Table 3). Additionally, the interview data showed that physiotherapists would give concurrent or feedforward information.

### Feedback Modality

Across therapists, unimodal auditory feedback (51.9%, SD 12.1%) or multimodal feedback with an auditory component was observed very frequently. The presentation of unimodal haptic feedback was an exception, with an average of 0.8% (SD 1.2%). The proportion of auditory or haptic feedback shows large interindividual differences (29.2%, SD 24.4%), that is, some participants used this modality very often, some did not use it at all.

Six participants emphasized that visual or auditory feedback should pertain to the initial starting position. In parallel, 4 participants highlighted the appropriateness of haptic feedback for guiding movement execution and indicating muscle activity.

### Integration of Content, Modality, and Timing

On average, terminal feedback was often coded in combination with the auditory subcategory for 18.2% (SD 5.1%). Simple confirming feedback was exclusively coded with the auditory modality for 28% across therapists, and the SD was relatively low, 8.5%. Conversely, the presentation of correct execution and elaborated feedback was coded in combination with auditory, auditory or visual, and auditory or haptic modality, and relatively large SDs were detected. Evaluative confirming feedback was almost equally coded with concurrent and immediate terminal feedback subcategories. The feedback that was coded as presentation of correct execution was often coded with the subcategory concurrent for, on average, 42.9% (SD 15.4%), and the same for elaborated feedback with 14.2% (SD 9.5%).

The interview data showed that there were single statements about when and how simple evaluative feedback should be presented. Unspecific confirmation was suggested as concurrent or terminal-delayed vibrotactile cues, while error signaling could be conveyed concurrently via vibrotactile, auditory, or visual channels. Correct execution feedback was exclusively described as concurrent and delivered through haptic, auditory, visual, or multimodal formats. Regarding elaborated feedback, comments were sparse. One participant recommended terminal-delayed feedback for addressing common errors, such as: “Yes, you could also show at the end that you had 90% of 100% correct posture. Or pay more attention to your abdominal tension in the next session because it needs a lot of stimuli now.” Additionally, multimodal concurrent or visual concurrent relevant hints could be presented about task-specific muscle activity (illustrated, for instance, by color-coded body regions).

### Feedback Localization

**Figure 6.**
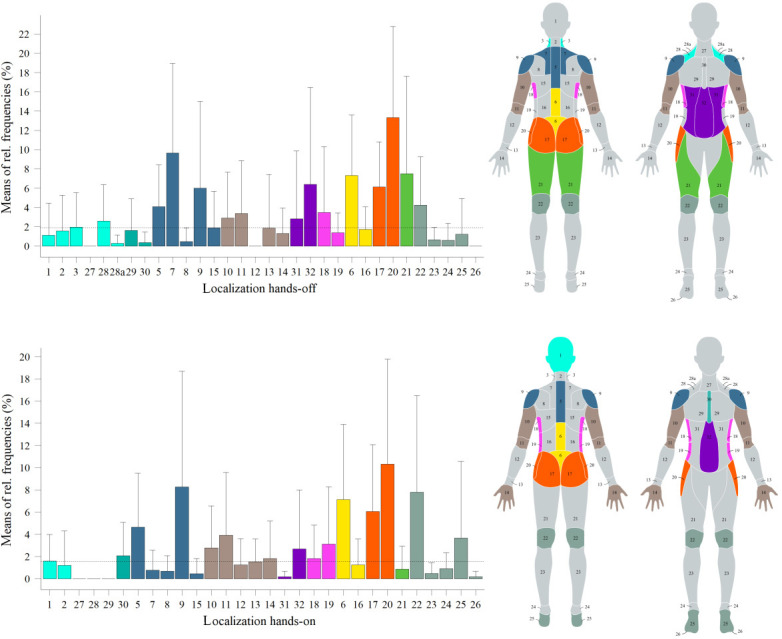
Mean relative frequencies (%) for body locations in hands-on and hands-off contexts during staged training, based on observed therapist-patient interactions; error bars indicate SD; values above the median (dotted line) are shown as colored areas on body diagrams. rel.: relative.

Figure 6 shows the means of the event-based relative frequencies (%) of individual body regions, both for the hands-on and the hands-off observation data.

Feedback on muscle activity was frequently associated with the abdomen and upper body, while movement execution feedback was linked to the shoulder, pelvis, gluteus, and arms. Starting position feedback was often related to the head, shoulders, thighs, lower legs, knees, feet, pelvis, gluteus, and lumbar spine. Evasive movements were predominantly associated with the pelvis, gluteus, and shoulders.

The interview data showed that half of the participants advocated providing vibrotactile feedback in specific anatomical regions, including the upper back or shoulder and lower back areas. An insightful suggestion emerged from 1 participant who underscored the necessity for individually tailored actuator settings in these regions, considering physical conditions such as kyphosis or lordosis tendencies and individual sensitivity to tactile stimuli in the scapula region. Additionally, 4 participants endorsed the application of vibrotactile cues to the thigh and gluteus or pelvis region. Opinions diverged among participants concerning feedback localization for the knee, leg, or foot, upper extremities, and neck or head regions. Notably, 2 participants opposed tactile feedback in the neck or head area.

Four of seven participants recommended the adaptability of localization to individual needs, enabling flexible actuator configurations based on physical attributes or personalized training progress. Three participants highlighted conceptual differentiation between static and mobile muscles during exercise execution, prompting the suggestion for diverse feedback forms (eg, variation in frequency) to accommodate these distinctions.

Feedback on starting position was associated with the upper extremities by 4 participants and with the lower extremities by 2. Muscle activity feedback was linked to the abdomen (3 participants), gluteus or pelvis (2 participants), lower back, and thighs.

## Discussion

### Foundations for Vibrotactile Feedback in Home Exercise Contexts

This paper aimed to derive requirements for a vibrotactile feedback strategy for home exercises. We gained initial insights into the provision of feedback and recommendations for a feedback strategy. Hypothesis-driven empirical studies based on initial approaches should follow. A strength of this work is the integration of a design-driven methodology with insights from both empirical and theoretical domains of instructional psychology.

### Feedback Content

The presentation of correct execution was frequently observed, and somewhat less regularly simple confirmatory feedback or elaborated feedback. Few participants recommended the presentation of elaborated information, and some recommended the use of presentation of correct results concerning different task requirements. For the design of the feedback strategy, deciding which task requirements should be covered in terms of content (eg, muscle activity, spatial movement accuracy, and velocity) seems relevant. The literature presents mixed results regarding the effectiveness of informational feedback, confirmatory feedback, or a combination of both in facilitating motor learning [39]. What content is effective depends on moderating factors such as task difficulty and skill level. The informational property of feedback becomes more important as task difficulty increases [40]. Especially for people with low skill levels, the presentation of information about solutions and ways of solving problems, as well as elaborated feedback, seems to be effective [41,42].

To design a feedback strategy for home exercising, it seems worthwhile to analyze the effects of feedback content on different learning states. Whether pain patients could benefit from elaborated feedback should be determined in further studies. Therefore, interaction effects should be considered. If, for example, elaborated feedback is combined directly with the presentation of the target performance, elaborated information has no additional effect [43].

The qualitative data can also identify reasons against the use of elaborated feedback. The participants mainly mentioned structural aspects such as the limited time frame of a treatment or the patients’ goal and motivation to become pain-free quickly, without wanting to achieve long-term learning effects. Further studies should examine the user acceptance and usability of the feedback strategy.

### Feedback Frequency

Participants recommended and showed reduced feedback frequency. They did not provide feedback for every movement repetition and error. This aligns with previous research linking improved motor performance with decreased feedback frequency during training and retention [44-47]. Conflicting results show no effects of reduced feedback as well as effects of dependency on task complexity, learning phase, and individual learner characteristics [2,45,46,48-50]. Physical therapists aimed to reduce system dependency and train proprioception. This also matches previous literature, which postulates that by reducing the frequency of feedback, the dependence on extrinsic feedback is reduced, and thus, intrinsic sources of feedback can be used [2,49]. Participants discussed reducing feedback frequency within a training session and over a more extended training period, suggesting the adaptability of the feedback strategy to both time frames. Externally controlled feedback was recommended, while self-controlled feedback was neither used nor suggested. The empirical literature does not clearly indicate a preferred technique for reducing feedback frequency [48,51].

Individual factors, such as susceptibility to errors, patient age, and physical condition, were identified as potential influences on feedback reduction. These recommendations are partly in line with previous research literature. For example, the optimal reduction in feedback frequency is related to the feedback recipient’s motor skill level and is influenced by perceived task difficulty [52]. For the design of a feedback strategy, adapting the feedback frequency to these parameters seems relevant.

### Feedback Timing

Results show that physical therapists provided frequently and unanimously recommended concurrent feedback. Delivering concurrent feedback can hinder learning, but it can support the performance of motor skills [17,48]. To apply a feedback strategy, feedback timing should be connected to its goal: performance enhancement or motor learning.

Additionally, the level of motor skills should be considered. Presenting concurrent feedback is initially beneficial for people with low motor skill levels [53]. So, there is a need to analyze the skill levels of patients.

Furthermore, physical therapists recommended the presentation of feedforward information. They suggested presenting information related to muscle activity or the starting position.

### Feedback Modality

Physical therapists frequently use auditory or multimodal feedback with an auditory component. This creates the need to translate or integrate verbal information into vibrotactile feedback. To translate verbal information, unimodal vibrotactile feedback can be presented with solid links to verbal information, for example, to movement directions in different planes. Literature describes this as a push or pull mechanism, moving the body away from the vibration or following the vibration [18,48]. Previous research shows individual preferences for intelligibility and the use of these mechanisms [18,54,55]. By activating multiple actuators, spatiotemporally bound vibration patterns can instruct movements [56].

Multimodal feedback can be used to integrate verbal information. This can cover the entire usage behavior that we found in our results (eg, some participants used certain modalities very often or very little). Literature also shows the benefits of providing multimodal feedback on motor performance and learning [15,57,58]. Our results show that visual or auditory information was connected to various task requirements. Six participants connected them to the correction of the initial position. Four participants connected tactile cues with spatial movement and muscle activity. To design a feedback strategy, it seems useful to conduct studies that investigate the advantages of unimodal auditory versus haptic versus multimodal feedback about initial position, spatial movements, and muscle activity.

### Integration of Content, Modality, and Timing

It was found that simple confirmatory feedback was mainly presented auditorily, concurrently, or terminally to a movement execution. The presentation of correct execution, as well as elaborated feedback, was mainly presented concurrently, in some cases terminally. To further develop the feedback strategy, we can define a type of feedback: confirmatory auditory feedback. It is then considered and needs to be studied whether this type of feedback differs from the presentation of correct execution and elaborated feedback.

### Feedback Localization

Feedback in the abdominal area was associated with muscle activity. Spatial motion accuracy was often coded with the pelvis, shoulder, or arm area. The starting position was frequently coded with head, shoulder, thigh, lumbar spine, pelvis, and knee. Thus, we see a tendency for feedback on muscle activity and spatial motion accuracy relating more to specific body parts than feedback on the starting position. A request for the feedback strategy could be to make vibrotactile cues distinguishable between muscle activity and spatial movements regarding the mentioned body areas.

Both in the hands-off context and from the interview data, haptic stimuli appear less set or intended on the forearm, hands, lower legs, and feet. Further studies should investigate whether auditory or visual information is sufficient to correct the positions of extremities.

### Limitations

This study has limitations that must be considered. First, the use of a healthy individual to demonstrate home exercises may limit the generalizability of our findings to patients with back pain, as patients may exhibit different movement patterns and errors [59,60]. We selected this approach to ensure exercise comparability and to guarantee the occurrence of comparable errors during the performance of the exercises. Having gained an overview of potential feedback behaviors, engaging in hypothesis-driven research on patient samples is a necessity. Additionally, the feedback behavior observed in this study may have been influenced by the presence of the experimenter and the use of cameras, which could have led to a socially desired behavior. Participants may have altered their actions to appear as correct as possible, potentially distorting the natural use of feedback. In some cases, the participants expressed that they were under pressure to act. Future studies should focus on real-world practice settings to minimize such biases.

Our study primarily focused on spatial accuracy, specifically whether participants’ movement paths aligned with the prescribed exercises. However, temporal factors [61], such as the number of repetitions, sets, and movement speed, were not considered in this study design. As a result, feedback related to these factors was rarely provided in the hands-on context. The interviews conducted in the hands-off context may not fully reflect physical therapists’ consideration of temporal factors, suggesting the need for future research that includes both spatial and temporal aspects in feedback strategies. Numerous factors influence whether home exercises are performed correctly, for example, memory capacity, motivation, attitudes, volition, or training conviction of the patients [61]. For designing an effective feedback strategy, the function of feedback should be differentiated. Further research is needed to determine how feedback can support the prescribed execution of home exercises.

### Practical Implementation

First, it should be noted that a feedback strategy should be flexible and adaptable to the patient. The present work suggests that a feedback system should consider the baseline of motor skill expression (eg, in the form of error frequency). The error thresholds should be set according to this baseline; thus, the feedback frequency can be influenced. Ideally, a feedback system can measure learning progress and adjust feedback frequency. In terms of feedback content, a language needs to be developed that patients can understand, both in terms of tactile presentation of the correct execution of exercise and in terms of confirmation of goal-directed action. Whether the presentation of elaborated information can increase the effectiveness of the feedback strategy needs to be investigated in subsequent studies. Although the focus of this paper is on a vibrotactile feedback strategy, the addition of auditory cues or visual information seems advisable. Suitable interfaces for the presentation of auditory and visual signals should be developed. Concurrent presentation of feedback is recommended. This feedback is presented vibrotactilely on muscle activity or spatial motion accuracy. For this purpose, actuators should be positioned primarily in the regions of the gluteus, pelvis, spine, deltoid, trapezius, abdominals, lateral to the trunk, thigh, and knee. Another implication for practice is the possibility of providing feedforward information on task-relevant musculature and the starting position.

### Conclusions

This work highlights that reduced feedback frequency, differentiated feedback content, multimodal feedback, and trunk-proximal vibrotactile feedback were recommended by physical therapists for a technical feedback system for the correct performance of home exercises. The results of this study provide an overview of relevant feedback characteristics and can serve as a starting point for further empirical research. Hypothesis-driven studies in sufficiently large patient samples should be followed up for the further development of a feedback strategy. Research-relevant topics include the perception, interpretation, and reaction of patients to vibrotactile feedback, as well as the integration of a technical-economic perspective regarding cost factors and hardware and software solutions.

## Supplementary material

10.2196/62903 Multimedia Appendix 1 Description of exercise execution.

10.2196/62903 Multimedia Appendix 2 Descriptions of category systems (hands-on and hands-off).

10.2196/62903 Multimedia Appendix 3 Detailed results of qualitative interview data hands-off.

10.2196/62903 Checklist 1 A 32-item COREQ checklist for qualitative interviews. COREQ: Consolidated Criteria for Reporting Qualitative Research.

## Abbreviations

COREQ: Consolidated Criteria for Reporting Qualitative Research
ITF: interactive tutoring feedback
