# Supplementary material for: Vibrotactile Feedback Strategies for Trunk-Stabilizing Exercises in a Home-Based Scenario: Qualitative Interview Study Among Physiotherapists
Source: JMIR Form Res. 2025 Jul 8;9:e62903. doi: 10.2196/62903 (PMC12262103; doi:10.2196/62903)

Multimedia Appendix 1

Table top

Target execution of the exercise: Knees are hip-width apart and hand-width off the floor. Hands are under the shoulders, head in line with the spine, back straight, pull shoulders away from ears. Establish basic tension, draw the belly button towards the spine, tense the gluteal muscles. Release the hand from the floor and rotate up with the spine. The pelvis is stabilized and does not tilt. Aim for maximum rotation. Rotate back at the same speed.

Figure S1. Visualization of correct execution of the exercise table top.

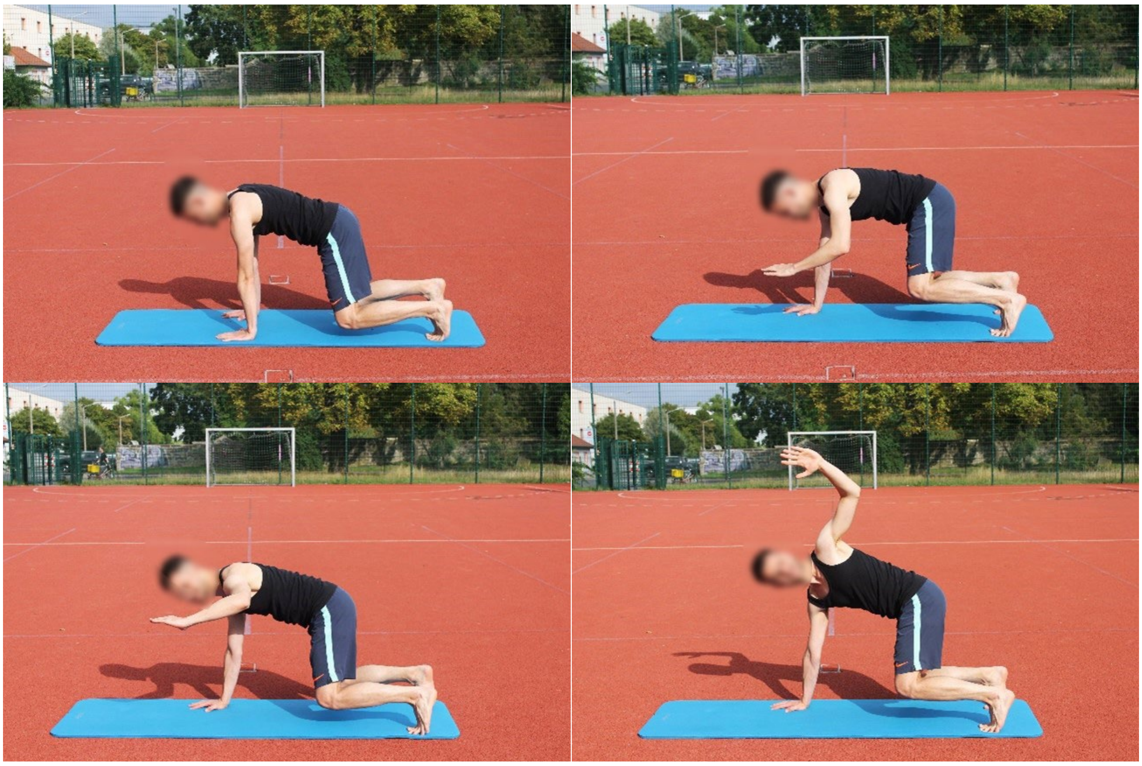

Two executions with typical errors:

1. Knees remain on the mat and are not lifted; the full range of motion is not utilized, the upper body only rotates up to a maximum of 90°.
2. Do not build up basic tension, this creates a hollow back; the pelvis rotates with the rotation.

Rowing

Target execution of the exercise: Feet are hip-width apart, knees are above the feet, heels are slightly raised so that you stand on the ball of your foot, knees are slightly bent. Upper body bent forward at an angle of 45°, back straight, head in line with the spine. Build up and hold basic tension. The arm pulls upwards, the shoulder blade pulls backwards and initiates the rotation. The arm is guided closely along the upper body. The pelvis is stable and does not rotate. Rotate back at the same speed.

Figure S2. Visualization of correct execution of the exercise rowing.

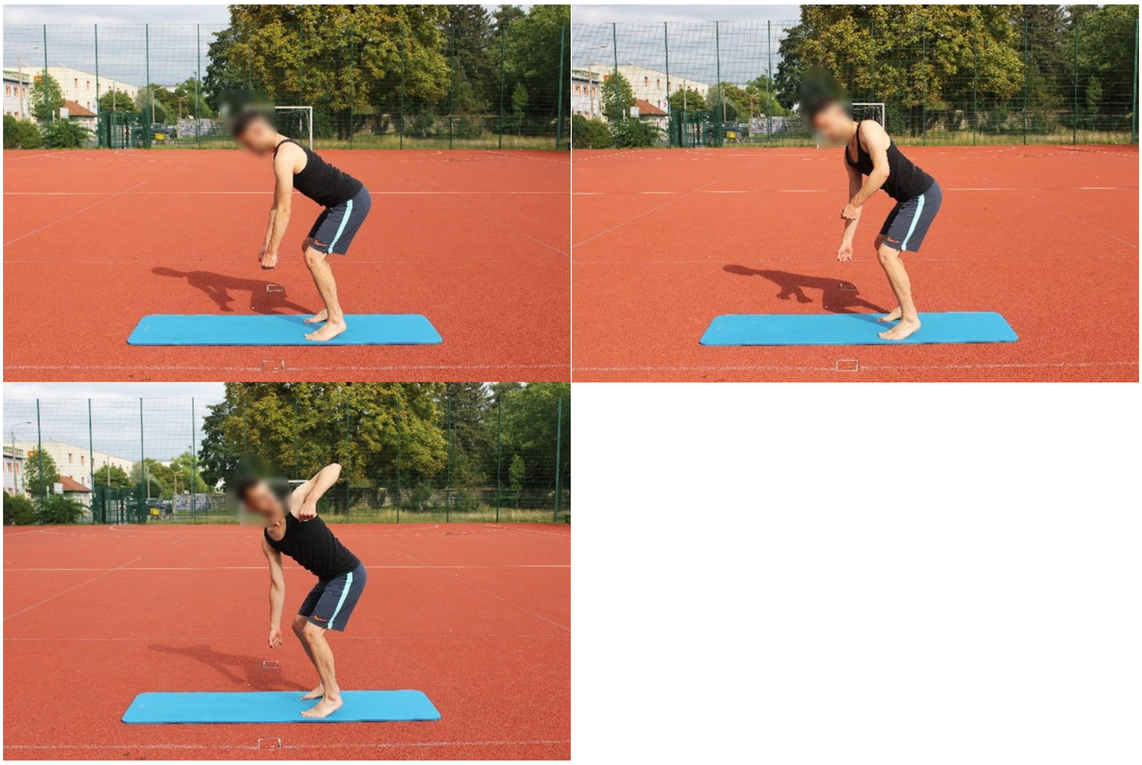

Two executions with typical errors:

3. Heels remain on the floor, knees are pushed through, upper body not leaning forward.
4. The arm is spread away from the upper body; the full range of movement is not used, maximum 45° rotation; basic tension is lost, and a hollow back develops.

Lateral plank

Target execution of the exercise: Shoulders, pelvis, knees, feet are in one plane. Elbow is positioned under the shoulder. The pelvis is raised to the maximum. The head is in the extension of the spine. Build up and hold basic tension. Press out of the shoulder with the supported arm. In this variation stabilise and hold the side support.

Figur S3. Visualization of correct execution of the exercise lateral plank.

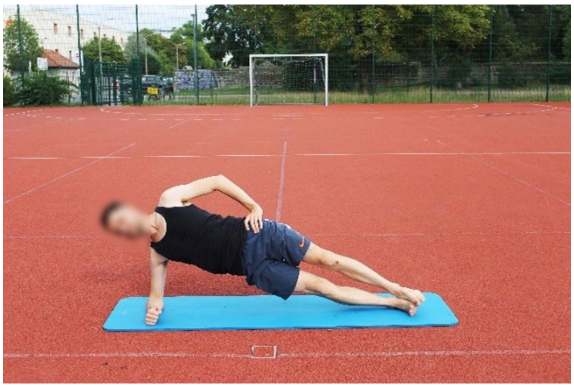

Two executions with typical errors:

5. The shoulder slumps. Shoulder, pelvis, knee are not on one level, the pelvis tilts backwards and forwards.
6. The elbow is positioned too far outwards and is not under the shoulder; the full range of movement is not used; the pelvis is only minimally lifted from the floor.

Squat

Target execution of the exercise: Arms are stretched at 90° from the upper body, the back is straight. Basic tension is built up and held. The head is in the extension of the spine. When bending the knees only come down so far that the heels do not come off the floor, go down with a straight back, do not let the knees move too much over the tips of the toes. Then push up again at the same speed.

Figur S4. Visualization of correct execution of the exercise squat.

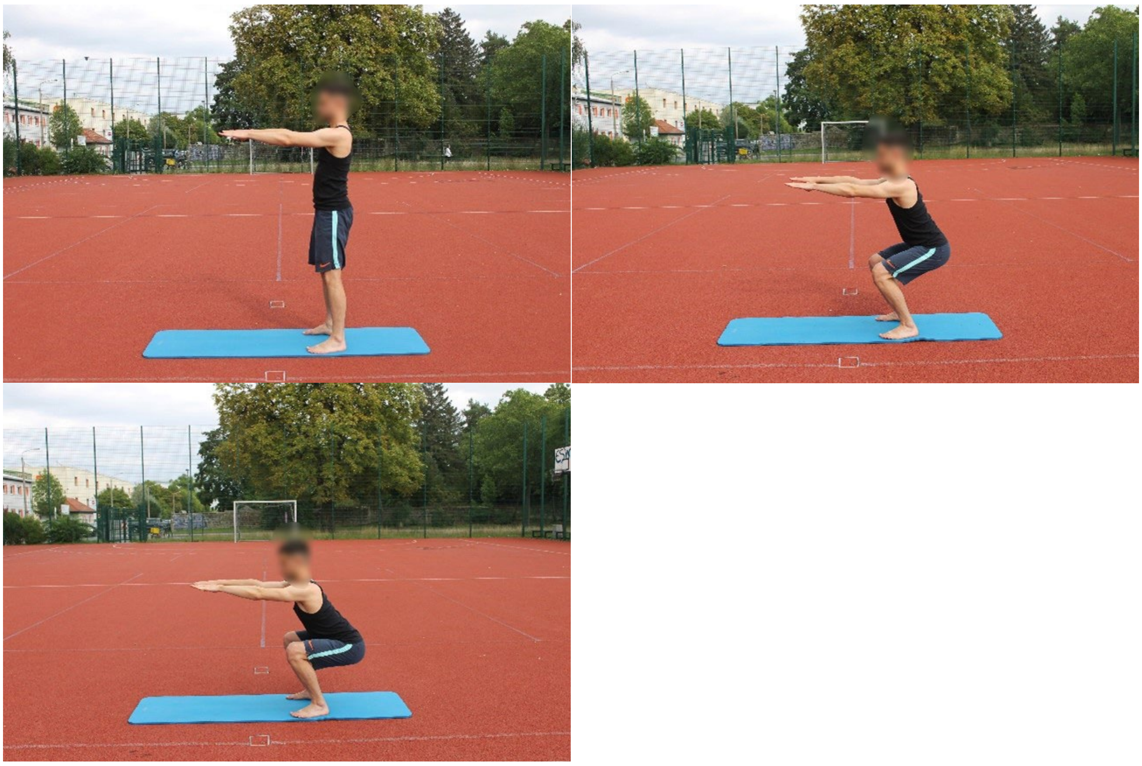

Two executions with typical errors:

7. The basic tension is lost, the upper back rounds and the chest sags; the knees move far beyond the tips of the toes.
8. The range of motion is not utilised, only slight bending of the knees; basic tension is lost, resulting in a hollow back.

Bridging

Target execution of the exercise: Feet and knees are hip-width apart. Place hands under the shoulders and pull the head into a slight double chin. Raise the pelvis while maintaining basic tension, then lower the pelvis slightly under tension. Tense the gluteal muscles.

Figure S5. Visualization of correct execution of the exercise bridging.

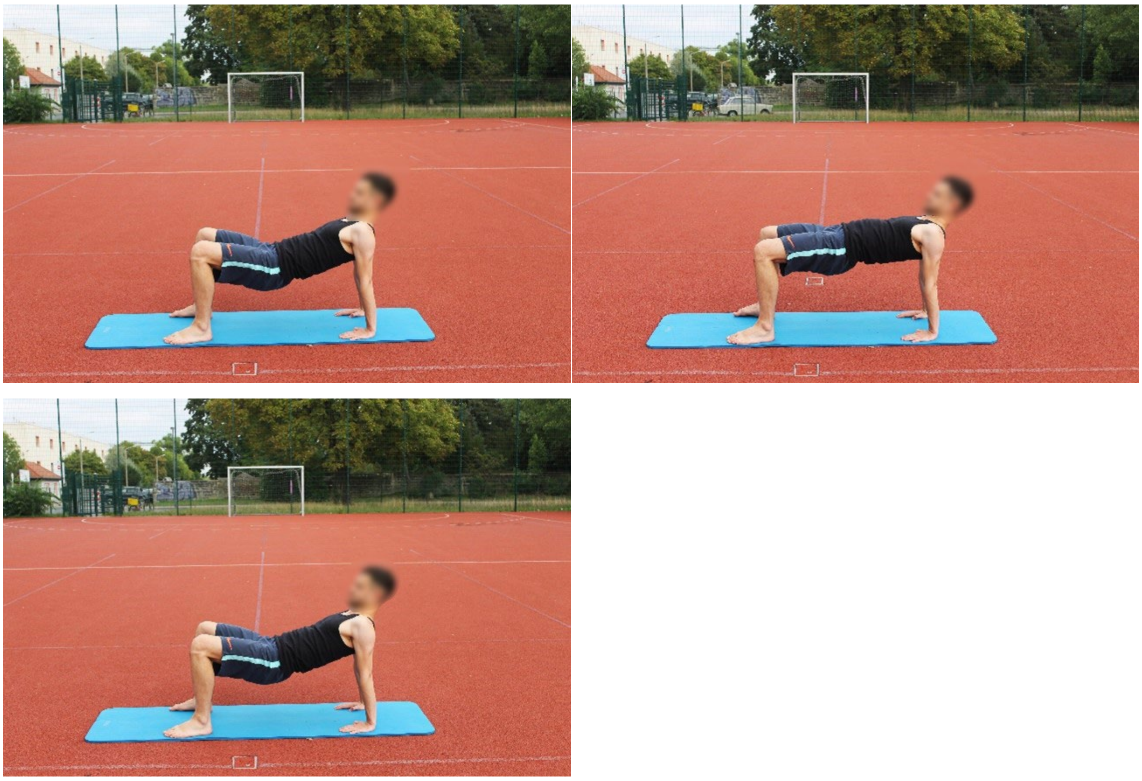

Two executions with typical errors:

9. Legs are very wide; the head hangs towards the floor.
10. The full range of movement is not used, the pelvis is moved only minimally; the arms are too wide.

Example design of training session

Figure S6. Procedure of training session, hands-on or hands-off. Number 1 to 10 are repetitions with errors, described above.

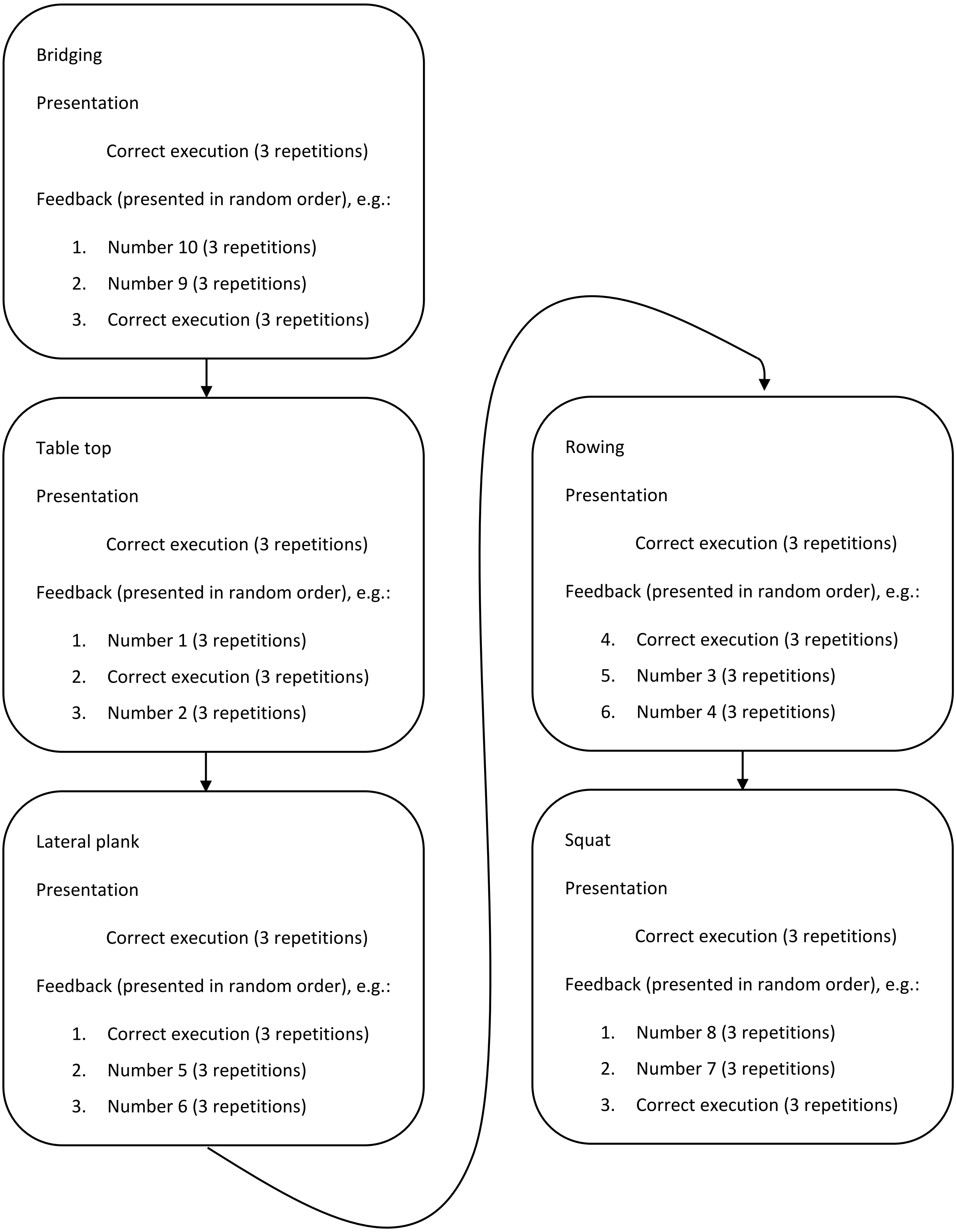

Supplement: Multimedia Appendix 1 [file formative-v9-e62903-s001.pdf]
