# Supplementary material for: Vibrotactile Feedback Strategies for Trunk-Stabilizing Exercises in a Home-Based Scenario: Qualitative Interview Study Among Physiotherapists
Source: JMIR Form Res. 2025 Jul 8;9:e62903. doi: 10.2196/62903 (PMC12262103; doi:10.2196/62903)
Supplement: Multimedia Appendix 2 [file formative-v9-e62903-s002.doc]

## Category system hands-on

### Coding segments for feedback frequency

The frequency of feedback was to be observed. The coded segments corresponded to the movement repetitions. Three movement repetitions per exercise execution were planned, but sometimes more or fewer repetitions were presented in the interaction, e.g. at the request of the participants. A movement repetition was the complete execution of the motion pattern of the exercise.

### Definition for feedback frequency

In the category frequency, a distinction was made whether feedback was given during or after a movement repetition or not. A movement element that followed feedback did not count as a movement repetition. If participants were still giving feedback and a movement followed in time, this movement did not count as a repetition. Only when the feedback was completed, and the entire movement pattern was repeated did this count as another repetition of the exercise within an exercise run. In the case of delayed or summative feedback, the feedback was counted as such, and the individual repetitions were counted as repetitions without feedback. A movement repetition was divided into the following subcategories:

1. Feedback

Definition: Feedback was given during a movement repetition or after the movement repetition with clear reference to it.

1. No feedback

Definition: A movement repetition for which no feedback was given was classified in this category. An "okay" given without additional comments did not count as feedback, as this was interpreted as a comment not intended for learning or performance growth.

### Coding segments for feedback properties

Only the behavioural sequences that contained extrinsic feedback were recorded using the following category system. Extrinsic feedback was defined as information given by participants to the stimulus subject as result of a behaviour that related to the current performance or learning status of the stimulus subject and could support further performance or learning. Multiple pieces of information were defined as one feedback if they related to the same behaviour, e.g. the same movement element or pattern, and occurred simultaneously. An example of extrinsic feedback was the touching of a body part (e.g. the pelvis) with the verbal information to change this body part in position (e.g. to push further up). If parts of a feedback were presented again within a movement repetition without offering complementary information, this was considered as a feedback at the time of the first information presentation. For example, if the participant first touched the pelvis with both hands for a position correction, held this touch with one hand, then corrected the shoulder position with the other hand and then brought the hand from the shoulder back to the pelvis, the tactile and verbal feedback related to the pelvis position counted as feedback at the time of the first pelvis touch, thus before the shoulder correction.

A feedback segment has been classified into the following main and subcategories.

#### Modality

Feedback was placed in this category based on the modality used to deliver it.

1. Auditive

Definition: Participants expressed information only in the form of spoken words or sounds.

Example: Participant said: "Please raise your head in line with your spine" without providing any additional visual or tactile stimuli.

1. Visual

Definition: Feedback was classified as visual if participants gave information to the stimulus person that the stimulus person could only perceive visually.

Example: The participant performs the intended movement him/herself, so that the stimuli person could observe it. The participant pointed to the relevant parts of the body.

1. Haptic

Definition: Feedback was classified as haptic if participants gave information only in the form of touch to the stimulus-de-person.

Example: The participant grips the pelvis of the stimulus-de-person and thereby initiates a tilting movement. The participant tapped on the shoulder of the stimulus-de-person.

1. Auditive-haptic

Definition: Feedback was classified as auditory-haptic if participants simultaneously used auditory and haptic stimuli related to a movement element or pattern.

Example: The participant touched the pelvis of the stimuli person and verbally instructed that the tailbone should be pushed towards the floor.

1. Auditive-visual

Definition: Feedback was classified as auditory and visual if participants provided auditory and visual stimuli simultaneously related to a movement element or pattern.

Example: The participant performed the intended movement him/herself so that the stimuli person could observe it and explained which muscle groups were relevant for this movement.

1. Auditive-haptic-visual

Definition: Feedback was classified as auditory and haptic and visual if participants simultaneously provided auditory and haptic and visual stimuli related to a movement element or pattern.

Example: The participant corrects the position of the knees by touching the knees with one hand and then using the other hand to illustrate the direction of movement of the knees while verbally explaining that the knees should not extend beyond the tips of the toes.

#### Localization

In the category localization, the body region to which a haptic feedback referred was defined more precisely. The subcategories were coded with 1 to 32. If haptic feedback could not be precisely assigned because it was not recorded due to the camera angle, the subcategory covered was used. If several body regions were touched, all of them were coded. Body regions are visualized (Figures S1 and S2).

**Figure S1.** Body regions, back view.


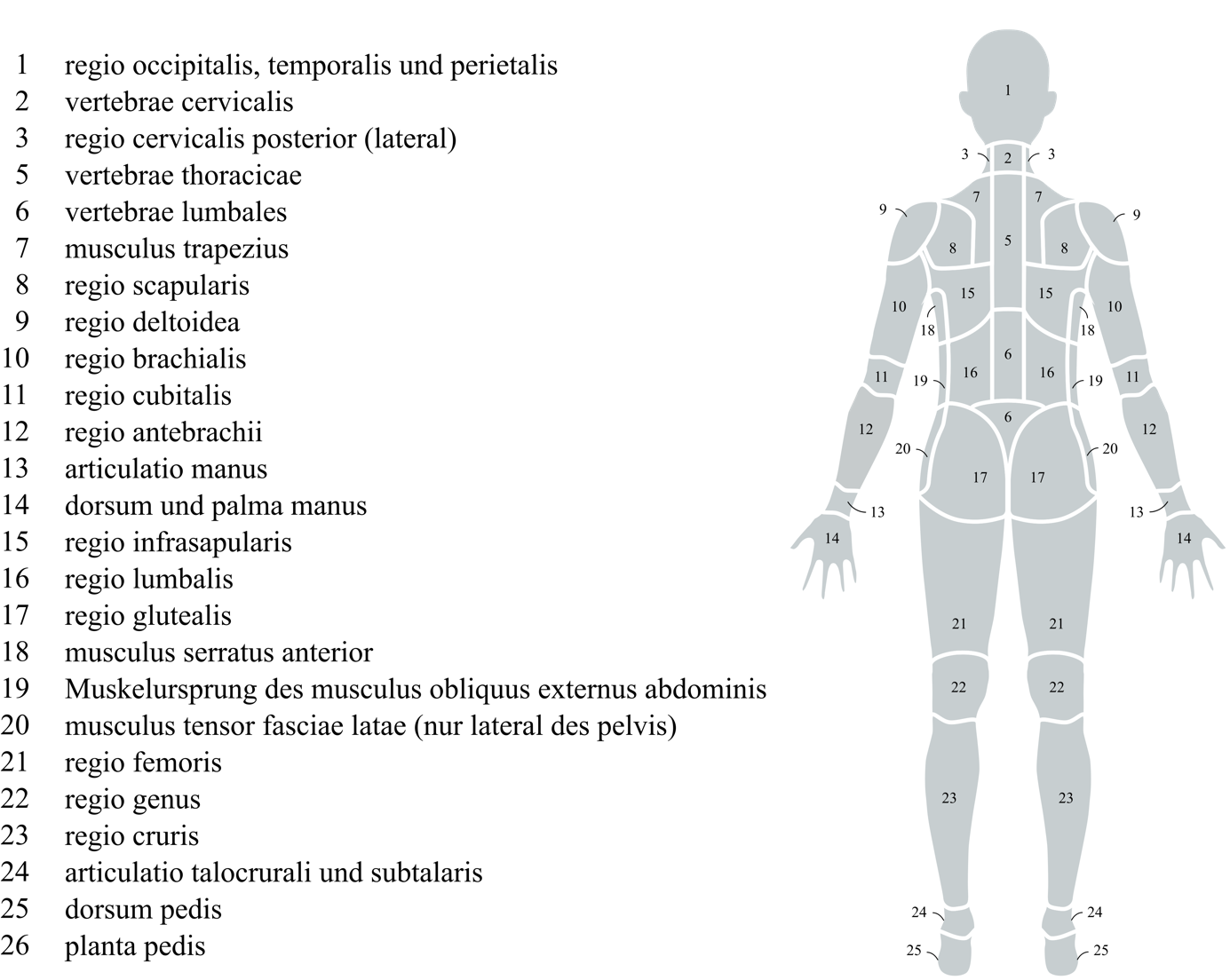


**Figure S2.** Body regions, front view.

**
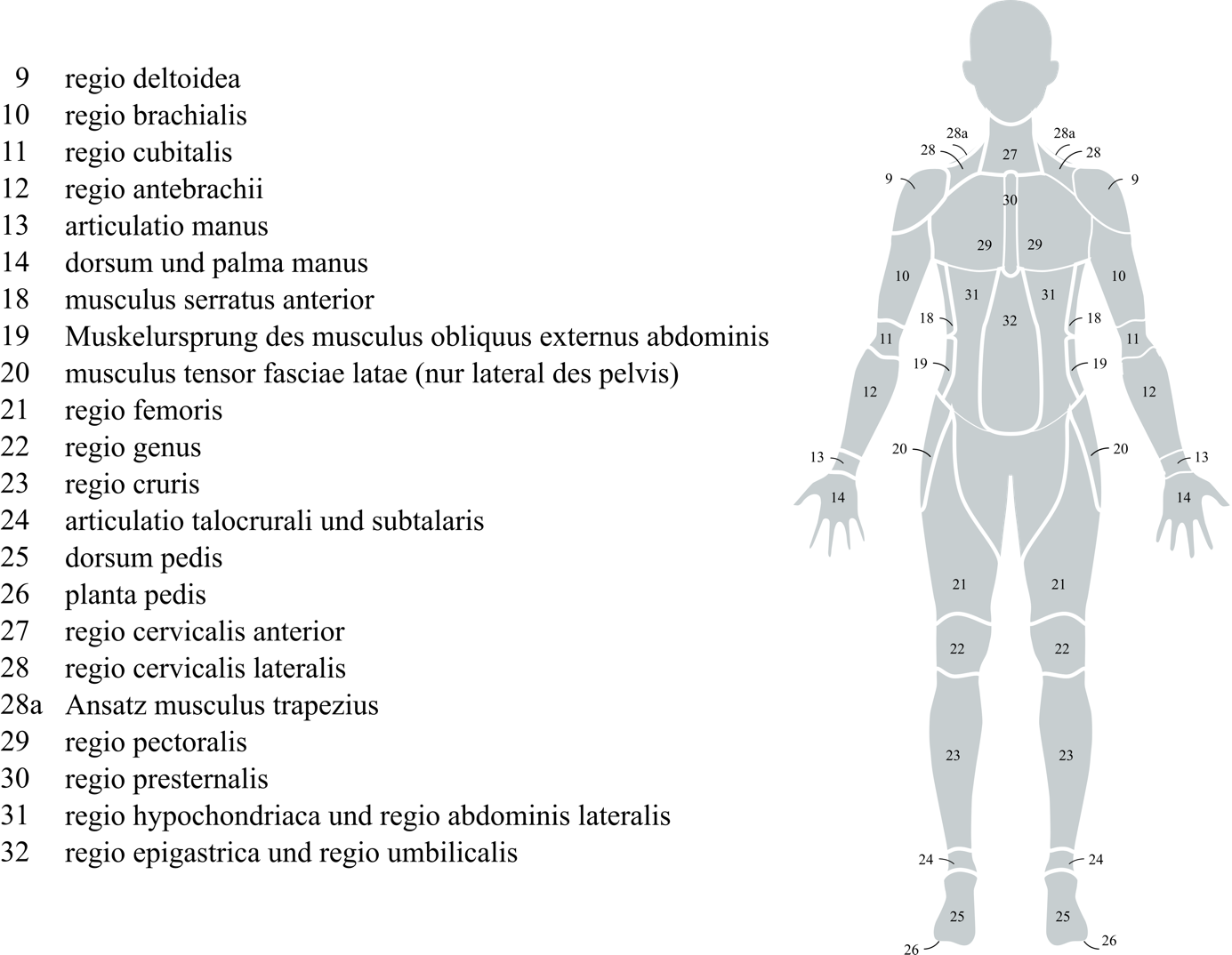
**

#### Time

In this category, the feedback was classified based on when it was given.

1. Concurrent

Definition: Feedback was categorised as concurrent if it was given during the performance of the behaviour to which it referred. Feedback that falls between movement repetitions and did not clearly refer to the first repetition is categorised as concurrent.

Example: The participant corrects the arm position while the stimuli person performs an arm movement.

1. Terminal (immediatly)

Definition: Feedback was categorised as direct terminal feedback if it was given after execution, immediatly following the behaviour to which it referred. If feedback was given following a movement/behaviour correction, it was categorised as final feedback if it clearly referred to the behaviour, even if it was given during further movement execution.

Example: Immediately after the completion of the execution of a position correction by the stimuli person, the participant confirms the behaviour with the words "Yes, it looks good like this.

1. Terminal (delayed)

Definition: The category of delayed final feedback includes feedback that was given with a time delay after the completion of the behaviour to which it referred. Feedback was only categorised as delayed if the feedback clearly referred to a behaviour that had occurred in the past, i.e. another behaviour had already occurred between the behaviour and the feedback.

Example: The participant describes errors of the first two repetitions only after the third repetition.

#### Content

1. Evaluative (confirmative).

Definition: Feedback exclusively contained information for the confirmatory evaluation of a movement pattern or movement element.

Example: The participant said: "That's good!”

1. Evaluative (rejection)

Definition: Participants exclusively expressed evaluative information that assumed incorrect behaviour.

Example: Participant said: "You haven't done that right yet!"

1. Presentation of correct execution

Definition: Feedback content was categorized as presentation of the correct execution when feedback contained information about the target movement or elements of the target movement. This could be exclusive content or could also contain additional outcome evaluation.

Example: The participant grabbed the pelvis and instructed the stimuli person to tilt the pelvis further forward to get into the correct position. The participant said: "Pull the ribs together".

1. Elaborated

Definition: The feedback content was categorised as elaborated information if additional information was provided beyond the evaluation or even without evaluative components. This could be hints on exercise execution, guiding questions, explanations, examples, communication of strategies or information on further execution of the task or descriptions of errors. Information on movement execution or muscle tension that was relevant for further repetitions and was formulated as such was categorised as elaborated information. If the information related only to a correction, it was categorised as evaluative information.

Example: participant said, "See what happens when your heels stay on the floor.", participant said, "Now when you go down, try to keep the extension of the lumbar spine."

#### Coding guideline hands-on.

The following scheme was used to systematically code the hands-on observations.

1. First mark the exercise sentences: Set 1,2,3. Check that each exercise consists of three sets.

2. frequency: code the feedback frequency for the movement repetition in a set (usually 3 repetitions per set).

3. if feedback was given: code modality, timing, and content.

4. if haptic feedback was given: code the localization

## Category system hands-off observations

### Coding segments

Touches that provided information for the use of vibro-tactile feedback systems and statements that related to the use of a vibro-tactile feedback system formed relevant observation units. Each observation segment was classified into the following main and subcategories:

#### Localization

In the category localization, the body region that the participants touched in the hands-off context was coded. Definitions and codes were taken from the hands-on context (Figures S1 and S2).

#### Task requirements

In the category task requirements, statements and observations were differentiated, which give indications of important components of the exercises.

1. Body position initial position

Definition: The category body position initial position described that information of a feedback referred to the initial position of static body segments and/or angular position of joints. Any feedback information given on the body position until the initial position was taken was coded as initial position. Information related to static elements after the initial position was taken was coded as evasive movements.

Example: The participant said, "I would do an [actuator] on the buttocks." And touched the stimuli person laterally on the pelvis. This feedback was given when the pelvis was not at the level of the target execution. The stimuli subject changed the pelvis position to the position of the target execution because of the feedback.

1. Body position evasive movements

Definition: The category of evasive movement referred to static elements of the exercise, i.e. a specific position of body segments and angular position of segments that changed during the exercise and deviated or threatened to deviate from the initial position. A movement/body position was categorized as an evasive movement after the initial position was taken for the first time,

Example: the stimuli person was already in the target position, the participant says: "I would put an [actuator] on the lower edge of the shoulder blade", touched the stimuli person at said position. This feedback could be observed after the target execution was already taken.

1. Body position movement execution

Definition: The category movement execution described that feedback referred to the body segments and/or angular position of joints during a movement, i.e. dynamic body segments.

Example: the participant said: "The elbow should be drawn towards the vibration."

1. Body position end position

Definition: feedback that referred to the position of body segments and/or angular position of joints reached after movement execution.

1. Muscle activity

Definition: Feedback related to the activation of specific muscles. Either this was explicitly mentioned, or statements were made about muscle tension or tension in certain parts of the body and the association between muscle activity and muscle activity.

Example: The participant described that he would put a vibration on the costal arch to make the patient contract the costal arch.

1. unspecific

Definition: Feedback segments that could not be coded with the subcategories already mentioned because no assignment was possible due to a lack of statements about a feedback segment were classified in this category.

## Category system hands-off interview

### Coding segments

Statements referring to the use of a vibro-tactile feedback system formed relevant observation units. To determine the segment length, only whole sentences were ever recorded as a segment; some segments extended over several sentences but were not shorter than one sentence. The segment started with the sentence containing information to be coded and ended with the sentence containing the last information of the content to be coded. The statements were given the codes of matching main and subcategories.

#### Modality

Statements about feedback modality were categorized in this category, statements were only coded if the modality was explicitly mentioned.

1. Auditory

Definition: In the auditory category, statements were classified that referred to feedback that should contain auditorily perceptible information, such as words, sounds or signals.

Example: "That he has to lower his arm further, that will be verbal, an instruction of the exercise must already be given."

1. Visual

Definition: The visual feedback category described information that participants recommended should be received visually.

Example: "The patient must already be able to check his starting position at home in the mirror."

1. haptic

Definition: The category haptic feedback described information that could be absorbed through the skin according to the participant's intention.

Example: the participant said: " [...] and I would actually just put a stimulus [...] put a stimulus on the belly and poof the belly button in."

1. multimodal

Definition: Statements that referred to more than one modality were coded into the multimodal category.

Example: The participant said that a tactile system had to be extended with a visual input, e.g. via a mirror.

#### Task requirements

In the category task requirements, statements and observations were differentiated, which contained information about the requirements of the exercises or behaviour on which feedback should be given.

1. Body position initial position

Definition: The category body position initial position described that information of a feedback referred to the initial position of static body segments and/or angular position of joints.

Example: The participant described that he would set a vibration at the pelvis to get the patient into the leg extension that corresponds to the initial position.

1. Body position evasive movements

Definition: Statements were coded with this category if feedback was to refer to static elements of the exercise, i.e. a certain position of body segments and angular position of segments that could change during the exercise.

Example: The participant mentioned that tactile feedback mainly works when it comes to nuances, when it comes to the need to stretch the back a bit more.

1. Body position movement execution

Definition: Statements were coded with this category if feedback should refer to body segments and/or angular position of joints during a movement.

Example: " [...] or I say, as for example in the pulling exercise, if the arm goes backwards here and he really can't manage it despite instruction, then I would tell him verbally, pull towards the vibration, you notice where the hand has to go, that he leads it there [...] ".

d. Body position end position

Definition: The category end position describes that feedback referred to the position of body segments and/or angular position of joints reached after movement execution.

e. Muscle activity

Definition: The category muscle activity described those statements referred to the activation of certain muscles. Either this was explicitly mentioned or statements about muscle tension or tension in certain parts of the body and the contraction of certain muscles were included in this category.

Example: "So the torso tension I would do before, [...] then you go into the starting position and at that moment there is the feedback for the torso [...]".

f. Endurance - Persistence in Exercise Performance

Definition: The category persistence included statements that refer to a continuous exercise execution within a training.

Example: The participant described that he would give feedback on the glutes, "especially when it goes into fatigue, to give another input, hey come on keep going".

g. Breathing

Definition: Statements were coded with this category if feedback was related to breathing.

Example: "Can you think of anything else [...] where you say, 'I can imagine with a tactile stimulus'"? - "Breathing definitely. [...]"

h. Velocity

Definition: The velocity category described that feedback referred to the velocity of exercise execution.

Example: The participant described that he would set an acoustic clock for the velocity of the exercise.

#### Localization

In the category localization, statements were included that referred to the use of feedback on specific parts of the body. Either the areas were explicitly named, or statements referred to more specific locations within an area.

1. Shoulder (regions: 5,7,8,9,15)
2. Chest (regions: 29,30)
3. Lower leg-knee-foot (regions: 22,23,24,25,26)
4. Thigh (regions: 21)
5. Pelvic-gluteus (regions: 17,20)
6. Arms (regions: 10,11,12,13,14)
7. Abdomen (regions: 31,32)
8. Lumbar spine (regions: 6,16)
9. Head and neck (regions: 1,1a,2,27,28,28a)
10. Upper body lateral (regions: 18,19)

#### Time

In the category of timing, statements were coded that contained information about the timing of the feedback.

1. feedforward

Definition: Participants stated that information should be given before the exercise is performed.

Example: "Basically, even if you say the big error pictures right away, maybe it's more like the exercise you're doing is generally announced again with the key points [...], if you include something like that at the beginning [...]".

1. concurrent

Definition: statements that referred to the feedback given during the execution of the exercise.

Example: "Directly, directly when a mistake is made, that he then corrects it immediately [...]".

1. Terminal feedback (immediately)

Definition: Participants stated that feedback should be given directly after the execution of the exercise.

1. Terminal feedback (delayed)

Definition: Participants stated that feedback should be given with a delay after the execution of the exercise.

Example: The participant said that he would give an affirmative praise signal to the patient at the end of an exercise (not after each set).

#### Content

1. Evaluative feedback (confirmative)

Definition: Statements that referred to the exclusive confirmation of correct behaviour by means of feedback.

Example: "When the starting position is set correctly, there should be a short vibration in the sense of 'that was now correct'".

1. Evaluative feedback (rejection)

Definition: Statements that referred exclusively to the evaluation of incorrect behaviour by means of feedback.

Example: "Several points say that there is something seriously wrong here, so that the exercise is stopped because of a massive error.

c. Presentation of the correct execution

Definition: Statements were categorized as presenting the correct outcome if feedback contained or was intended to contain the intended target movement or elements of that target movement. This could be exclusive content or also contain additional outcome evaluation.

Example: "Either I use the tactile feedback to say, 'I need more activity here', which is, for example, the one on the buttocks, [...]"

d. Elaborated information

Definition: Statements about the feedback content were categorized as elaborated information if participants recommended providing additional information for evaluation, this could be hints about exercise execution, guiding questions, explanations, examples, providing strategies or information for further execution of the task, and descriptions of errors.

Example: "Exactly, something that is not yet explicit on a body part, but something that says, 'you deviate from the optimal variant, try something else and see how it reacts then'.

#### Frequency

The frequency of feedback described a temporal aspect of giving feedback and defined the number of feedback sequences intended by the participants.

1. Constant

Definition: The subcategory constant described that feedback should be given on every intended event.

Example: The participant stated that feedback should always be given when a certain error occurs.

1. Reduced - externally controlled

Definition: The subcategory reduced - externally controlled described that participants recommended to reduce the frequency of feedback. The reduction in frequency should be controlled by the system. For example, the frequency of feedback could be reduced with increasing learning success or feedback could only be offered in a proportion of training units.

Example: The participant stated that there should be less feedback after 3 or 4 weeks, for example, so that "you don't have to train with it all your life".

1. Reduced - self-controlled

Definition: Statements were assigned to the category reduced self-controlled if they referred to the fact that patients should be able to request their feedback independently.

Example: The participant described that the system should be able to be switched on and off in a modular way.

d. Reinforced

Definition: Statements in the reinforced category described phases/times when feedback should be reinforced.

Example: The participant described that especially at the end of a repetition/exercise, "when it gets tiring, [to give] input again [...] so that he doesn't get sloppy.”

#### Function

In the category feedback function, it was discriminated which functional level participants intended to address with a feedback system.

1. Motivational

Definition: The motivational category included statements that feedback should have an effect on a motivational level. This was either stated directly or statements contained information about activating the subjective experience of competence, reducing task difficulty, increasing the subjective probability of success or the internal attribution of success.

Example: The participant stated that feedback should have a certain motivating factor.

1. Strategy acquisition to strengthen intrinsic feedback

Definition: This category included statements that feedback on strategy acquisition should support one's own body awareness.

Example: The participant stated that a vibration should remind a person to check their body position.

1. Gaining knowledge

Definition: Statements were categorized as having an effect on the cognitive level if they referred to informative feedback intended to expand, erase, change or restructure knowledge.

Example: "And possibly here between the shoulder blades, so that he knows, okay I am actively turning up from the shoulder blades.

d. Improving performance through guidance

Definition: This category captures statements that feedback should only aim to support or improve current performance.

Example: The participant mentions that feedback should be used in a corrective way to explicitly guide movements.

#### Individual parameter

1. Understanding of the exercise

Definition: The category understanding of the exercise included statements that the design of the feedback system is influenced by the understanding of the participants. And that the ability to perform key elements of the exercise (starting position of the trunk, extremities, head, and basic movement execution) must be given.

1. Information processing skills - Feedback Interpretation

Definition: Statements were placed in this category if they contained information to translate the feedback.

Example: The participant talked about different types of patients either understanding vibration as a cue for muscle activation or as a support in movement execution.

1. Information processing skills - feedback perception

Definition: Statements were categorized as feedback perception if they contained information about customizable frequencies or vibration patterns.

Example: The participant mentioned that there are individual tolerances for the acceptance of frequencies.

1. Exercise motivation

Definition: Statements were placed in the exercise motivation category if they contained information that an individual exercise or training motivation should be considered.

1. Physical condition

Definition: Statements were placed in the category of physical conditions if they referred to the fact that the patient's physical condition, specific anatomy, physical fitness, or the patient's specific medical condition have an influence on the feedback design.

Example: "If the maximum kyphosis is quite high, that is individual, then I do not need to give tactile feedback there.

1. Level of motor skills

Definition: Statements were coded with this category if they referred to the influence of the different motor skills of patients on the design of extrinsic feedback. The level of competence was defined by the ability to perform exercises of different degrees of complexity and the proportion of potential errors.

Example: The participant described that the feedback frequency depends on the patient's susceptibility to errors and that this must be considered in the calibration.
