# Supplementary material for: Vibrotactile Feedback Strategies for Trunk-Stabilizing Exercises in a Home-Based Scenario: Qualitative Interview Study Among Physiotherapists
Source: JMIR Form Res. 2025 Jul 8;9:e62903. doi: 10.2196/62903 (PMC12262103; doi:10.2196/62903)
Supplement: Multimedia Appendix 3 [file formative-v9-e62903-s003.docx]

**Table S1**. Feedback properties named by physiotherapists in interviews, grouped by theme and linked to task requirements and individual parameters; in brackets: number of participants mentioned the topic.

| Feedback properties | Themes | Task requirements | Individual parameter |
| --- | --- | --- | --- |
| **Content** | | | |
| Evaluative | Unspecific confirmation (3) |  |  |
|  | Specific confirmation (1) | Spatial movement execution (1) |  |
|  | Unspecific error signaling (2) | Initial position (2) |  |
|  |  | Spatial movement execution (1) |  |
|  | Presentation of correct execution (8) | Muscle activity (4) | Feedback interpretation (2) |
|  |  | Spatial movement execution (4) |  |
|  |  | Initial Position (3) |  |
|  |  | Motion velocity (2) |  |
|  |  | Persistence in training/repetitions (2) |  |
| Elaborated | Cues for self-monitoring (1) |  |  |
|  | Supporting try and error (1) |  | Feedback perception (1) |
|  | Presenting task relevant hints (3) | Initial Position (1) |  |
|  |  | Muscle Activity (2) |  |
|  |  | Breathing (1) |  |
|  | Summative error feedback (1) |  |  |
| **Frequency** | | | |
| Reduced | Fading within training period (6) | Persistence in training/repetitions (1)  Muscle activity (2) | Level of motor skills (1)  Physical condition (Age, Diseases) (4) |
|  | Fading within single training session (2) |  |  |
|  | Increasing within single training session (4) |  |  |
|  | Self-controlled |  | Individual motivation for exercising |
| **Timing** | | | |
| Feedforward |  | Initial Position (3) |  |
|  |  | Muscle activity (3) |  |
| Concurrent |  | Initial Position (1) |  |
|  |  | Muscle activity (3) |  |
|  |  | Spatial movement execution (1) |  |
| **Modality** | | | |
| Visual or auditive |  | Initial position (6) |  |
|  |  | Evasive movements (1) |  |
|  |  | Spatial movement execution (1) |  |
|  |  | Persistence in training/repetitions (1) |  |
|  |  | Motion velocity (1) |  |
| Haptic |  | Initial position (1) |  |
|  |  | Evasive Movements (1) |  |
|  |  | Spatial movement execution (4) |  |
|  |  | Muscle activity (4) |  |
| Multimodal |  | Evasive movements (1) |  |
| **Body Localization** | | | |
| Shoulder – Upper back |  | Evasive movements (1) | Physical condition (anatomy) (1) |
|  |  |  | Feedback perception (1) |
| Lower back |  | Muscle activity (1) | Physical condition (anatomy) |
|  |  | Evasive movements (1) |  |
| Gluteus – Pelvis |  | Muscle activity (2) |  |
|  |  | Spatial movement execution (1) |  |
| Tigh |  | Muscle activity (2) |  |
|  |  | Initial position (1) |  |
| Knee – Leg – Foot |  | Initial position (4) | Physical condition (anatomy) (1) |
|  |  |  | Feedback perception (1) |
|  |  | Spatial movement execution (1) |  |
| Arm – Hand |  | Initial Position (4) |  |
|  |  | Spatial movement execution (1) |  |
| Neck – Head |  |  |  |
| Abdomen |  | Muscle activity (3) | Feedback perception (1) |
| Unspecific |  |  | Physical condition (weight, Diseases) |
