# Supplementary material for: Vibrotactile Feedback Strategies for Trunk-Stabilizing Exercises in a Home-Based Scenario: Qualitative Interview Study Among Physiotherapists
Source: JMIR Form Res. 2025 Jul 8;9:e62903. doi: 10.2196/62903 (PMC12262103; doi:10.2196/62903)
Supplement: Checklist 1 [file formative-v9-e62903-s004.docx]

**Table S1.** COREQ checklist 1.${}^{a}$

| No | Item | Description | Answer (paragraph in manuscript) |
| --- | --- | --- | --- |
| 01 | Interviewer/facilitator | Which author/s conducted the interview or focus group? | Master's student at the Department of Psychology of Learning an Instruction |
| 02 | Credentials | What were the researcher’s credentials? | Bachelor of Science, trained in qualitative research |
| 03 | Occupation | What was their occupation at the time of the study? | Master's student |
| 04 | Gender | Was the researcher male or female? | female |
| 05 | Experience and training | What experience or training did the researcher have? | Methods training |
| 06 | Relationship established | Was a relationship established prior to study commencement? | no |
| 07 | Participant knowledge of the interviewer | What did the participants know about the researcher? | Nothing, apart from working for TU Dresden |
| 08 | Interviewer characteristics | What characteristics were reported about the interviewer/facilitator? | no characteristics |
| 09 | Methodological orientation and Theory | What methodological orientation was stated to underpin the study? | Theoretical: ITF Model / instructional psychology and HCD (80-94; 114-119; 183-185)  Systematic data analysis (243-247) |
| 10 | Sampling | How were participants selected? | Purposive sample (147 – 149) |
| 11 | Method of approach | How were participants approached? | Telephone, Email (147-148) |
| 12 | Sample size | How many participants were in the study? | 30 public physiotherapy facilities – 9 facilities participated (147-148) |
| 13 | Non-participation | How many people refused to participate or dropped out? Reasons? | 21 refused, reasons: no answers, no interest, no time |
| 14 | Setting of data collection | Where was the data collected? | Dresden; at the participants’ facilities (205-206) |
| 15 | Presence of non-participants | Was anyone else present besides the participants and researchers? | no |
| 16 | Description of sample | What are the important characteristics of the sample? | Demographics, occupation and working experience see Table 2 |
| 17 | Interview guide | Were questions, prompts, guides provided by the authors? Was it pilot tested? | Interview guide development grounden in the ITF model, see Table 1;  Study setting was pilot tested (195-203) |
| 18 | Repeat interviews | Were repeat interviews carried out? If yes, how many? | no, only 1 interview per participant |
| 19 | Audio/visual recording | Did the research use audio or visual recording to collect the data? | Both (190-191; 237-238) |
| 20 | Field notes | Were field notes made during and/or after the interview or focus group? | field notes were made (191-192) |
| 21 | Duration | Duration What was the duration of the interviews or focus group? | Interview: 15 minutes (190); full study 90 minutes |
| 22 | Data saturation | Was data saturation discussed? | Data saturation was reached during coding process, as no new subcodes were identified |
| 23 | Transcripts returned | Were transcripts returned to participants for comment and/or correction? | no, due to time limitation; |
| 24 | Number of data coders | How many data coders coded the data? | same coder repeated trial coding; discussion with team member from research team (253) |
| 25 | Description of the coding tree | Did authors provide a description of the coding tree? | yes, Figure 4-6 |
| 26 | Derivation of themes | Were themes identified in advance or derived from the data? | deductive and inductive coding (246-250) |
| 27 | Software | What software, if applicable, was used to manage the data? | MAXQDA Plus 2022 (Release 22.2.0) |
| 28 | Participant checking | Did participants provide feedback on the findings? | no, due to time limitation; the analysis was conducted with close attention to accurately reflecting their views. |
| 29 | Quotations presented | Were participant quotations presented to illustrate the themes / findings? Was each quotation identified? | yes |
| 30 | Data and findings consistent | Was there consistency between the data presented and the findings? | Findings were derived from a combination of interview and observational data. Interpretations in the discussion were grounded in both data sources, and representative quotes and examples from observations were used to illustrate and support the key themes. |
| 31 | Clarity of major themes | Were major themes clearly presented in the findings? | Feedback characteristics are presented separately as well as integrated, based on observational findings and interview data. |
| 32 | Clarity of minor themes | Is there a description of diverse cases or discussion of minor themes? | contrasting individual perspectives and less frequently occurring themes, from which future research questions emerge, were addressed; the diversity of perspectives was also demonstrated. |

${}^{a}$items and desciptions from the 32-item COREQ checklist, extended with answers

##

## References

1. Tong A, Sainsbury P, Craig J. Consolidated criteria for reporting qualitative research (COREQ): a 32-item checklist for interviews and focus groups. Int J Qual Health Care. Dec 2007;19(6):349-357. doi:10.1093/intghc/mzm042
